# What's the point of the *BJPsych Bulletin*?

**DOI:** 10.1192/bjb.2018.25

**Published:** 2018-06

**Authors:** Norman Poole

**Affiliations:** 1 South West London and St Georges Mental Health NHS Trust

## Abstract

The incoming editor Dr Norman Poole sets out a vision for his tenure at the helm of the *BJPsych Bulletin*. There will be redoubled focus on training and educational articles and an engagement with the cultural and social ramifications of this most vital of specialisms.

**Declaration of interest:**

Dr Poole is editor of the *BJPsych Bulletin*.

I am honoured and not a little trepidatious taking over the reins (or reign) of the *BJPsych Bulletin* from departing editor Jonathan Pimm. Earlier in his career, Jonathan was a *bona fide* newspaperman and clearly relishes the business, since he is now moving on to *The Lancet* to take up the role of associate editor. During his editorship of this publication, Jonathan introduced open access in pursuit of egality, FirstView online for efficiency, and fewer briefer editions for expediency. He is a hard act to follow, so I won't even try. I would, however, like to thank him for his work on the *Bulletin* over his 5-year tenure and to wish him all the best in his future endeavours.

While preparing for the interview for this post, I discovered that the *Bulletin* aspires to be ‘the foremost source of information about all aspects of mental healthcare’. A high bar indeed. In practice, its articles predominantly cover education, service provision, op-eds and profiles of renowned psychiatrists, both living and recently deceased, plus a smattering of book reviews and letters. While the *Bulletin* is seen as a place to publish original studies about service delivery, we are not here to publish research in the sciences basic to psychiatry. A glance at the most-read articles on the website suggests our readers value this focus, so I'm mindful that this should not be lost sight of in any future revisions. Still, it's the new guy's prerogative to tinker, and so I feel compelled to shape the *Bulletin* in my own image; but what sort of image is that?

While I ought to have been reading cardiology textbooks, I was in fact nose-deep in Edward O. Wilson's *Sociobiology*,^1^ a fact that was to be reflected equally in my final exam scores and the ensuing purchase of Stephen Rose's edited volume *From Brains to Consciousness?*^2^ I diverted myself from the former circumstance with Tim Crow's chapter claiming an intimate link between language development and schizophrenia, alongside Richard Bentall's, which questioned the validity of diagnosis. I found the stark divergence of explanatory theories and dispute about the very grounds of the debate invigorating after 5 years of didactic learning, so decided there and then to become a psychiatrist. Much as I admire my cardiology colleagues – and if I ever have heart trouble, I'd like to see one who doesn't doubt its existence – nothing else in medical school compared to the dizzying exciting uncertainty of the science(s) of human behaviour.

Of course, psychiatry also interacts with culture and values, more so than other medical specialities. I still firmly believe we are incredibly fortunate and privileged to work in such an intellectually stimulating and diverse, even at times fragmented, field. Yet, it appears to me that there is nowhere obvious other than the *Bulletin* for essays and articles that deal with psychiatry's myriad relationships. For instance, proposed changes to the Mental Health Act, the representation of psychiatry in the arts and media, philosophical and cultural critiques of psychiatric concepts, and so forth. The intention is not to criticise psychiatry but for the *Bulletin* to be a place for genuine reflection, which occurs within the profession and specialist journals but is not always easily accessible or visible to trainees. I'd like to strengthen the *Bulletin*'*s* coverage of psychiatry in all its breadth and glory.

Although the *Bulletin* should not concern itself with basic neuroscience, a core function is training and education. As Professor Wendy Burn has identified (http://www.rcpsych.ac.uk/discoverpsychiatry/pastpresidentsblog/neuroscienceincurriculum.aspx), learning about the neuroscience that underpins psychiatry is an area trainees find challenging. The Gatsby Foundation is currently partnering with the Royal College of Psychiatrists to review the training curriculum for neuroscience, and I hope to support this initiative by using the *Bulletin* as a forum for discussion and dissemination. I encourage authors to submit clinically relevant and readable reviews of neuroscience topics, such as Nour & Nour's recent paper on visual hallucinations.^3^

Finally, I believe that psychiatry trainees (and non-training grades) would benefit from a specific section similar to the *BMJ*'s *Endgames* format exploring complex psychiatric presentations and management issues. I envisage that this section, to be co-written by consultants and trainees, will support the development of clinical reasoning,^4^ which is often hard to fathom for trainees in a busy clinic. In particular, the series should focus on differential diagnosis (identifying or excluding ‘organic’ aetiology; differentiating between superficially similar conditions such as adult attention-deficit hyperactivity disorder and borderline personality disorder). I encourage prospective authors to contact the *Bulletin* with proposals for more detailed advice on guidelines. Do please note that standards must accord with the International Committee of Medical Editors' Uniform Requirements for Manuscripts (http://www.icmje.org/) and co-production with patients is encouraged.

So, more op-ed pieces on the state of psychiatry and a focus on trainees' needs around neuroscience and clinical complexity. Is it achievable? Like all journals, whether the *Bulletin* sinks or swims depends on the articles submitted for consideration. I take this opportunity as your new editor to encourage readers to consider what you'd like the *Bulletin* to be and to get writing. It is, in fact, not my image that will shape the *Bulletin*, but yours.

## About the author

**Norman Poole** is Editor of the *BJPsych Bulletin* and Consultant Neuropsychiatrist at St George's Hospital, South West London and St George's Mental Health NHS Trust, London, UK.
